# The Challenges and Opportunities for Mental Health Twin Research in Nigeria

**DOI:** 10.1007/s10519-023-10153-y

**Published:** 2023-09-21

**Authors:** Olakunle Ayokunmi Oginni, Ayoyinka Ayorinde, Kehinde Dorcas Ayodele, Onyedikachi Joseph Opara, Boladale Mapayi, Kolawole Mosaku

**Affiliations:** 1 Department of Mental Health, Obafemi Awolowo University, Ile-Ife, Nigeria; 2 Department of Mental Health, Obafemi Awolowo University Teaching Hospitals Complex, Ile- Ife, Nigeria; 3 Social, Genetic and Developmental Psychiatry, King’s College London, London, UK

**Keywords:** Twin research, Genetics, Nigeria, Sub-Saharan Africa, Low- and Middle-Income Countries, Patient Participant Involvement and Engagement (PPIE)

## Abstract

The recent interest in increasing diversity in genetic research can be useful in uncovering novel insights into the genetic architecture of mental health disorders – globally and in previously unexplored settings such as low- and middle-income settings like Nigeria. Genetic research into mental health is potentially promising in Nigeria and we reflect on the challenges and opportunities for twin research which may be particularly suited to Nigeria. The higher rates of twinning in Africa and Nigeria specifically, make the twin design an affordable and readily maintainable approach for genetic research in the country. Despite potential challenges with recruitment, data collection, data analysis and dissemination; the success of current efforts suggest that the twin design can tapped even further for greater impact in the country. We highlight some ways in which the scope of twin research can be increased and suggest some ways in which existing challenges can be overcome including recent Patient Participant Involve and Engagement activities.

## Background

Mental health in low/middle-income countries (including Nigeria and most of sub-Saharan Africa) is under-researched and under-resourced (Owen et al. 2016; Sankoh et al. 2018). This is despite the high mental health burden and high unmet need – up to 1 in 8 Nigerians meet the diagnostic threshold for psychiatric disorders (Gureje et al. 2006). Of these, only 8% receive treatment (Gureje et al. 2006) which partly reflects the limited mental health workforce in the country (Nwaopara 2015). The high population in Nigeria (estimated at 213 million in 2021; The World Bank) indicates a large absolute number of people living with mental health difficulties. Considering the high unmet mental health need, a cost-effective intervention should focus on prevention. However, such efforts can only be informed by high quality research. Unfortunately, there is a dearth of rigorous and systematic research into the etiology of psychiatric disorders in sub-Saharan Africa (Owen et al. 2016). Mental health research in Nigeria needs to reflect the complex etiology of psychiatric disorders by investigating genetic and environmental influences on mental health risk and interplays between these.

### Genetic Research in Nigeria

Until the present time, most genetic research in Nigeria has focused on physical conditions such as sickle cell disease, chromosomal disorders (Adeyemo et al. 2018) and malignancies (Alatise et al. 2021). These have been useful to inform interventions such as premarital checking of hemogblobin genotypes to reduce the likelihood of sickle disease while the relatively different genetic signature of colorectal cancers among Nigerians highlights the need for more region-specific research (Adeyemo et al. 2018; Alatise et al. 2021). The evidence base for etiological mechanisms of mental health problems has typically been adopted from research findings in higher-income countries. However, the interplay of these influences may not necessarily be the same in low- and middle-income versus higher-income settings (Oginni & Hur, 2022). In recognition of this and the need for greater diversity in genetic research (Martin et al. 2022), there are increasing efforts to increase participation of low- and middle-income non-Western countries in genetic research. Such efforts include the Neuropsychiatric Genetics of African Populations Psychosis (NeuroGAP-Psychosis) project and the Global Initiative for Neuropsychiatric Genetics Education in Research (GINGER) (Martin et al. 2022). The NeuroGAP-Psychosis project aims to boost diversity in genetic research by recruiting participants from Africa. To complement this, the GINGER is focused on building expertise in genetic research among researchers based in Africa. Though these projects are improving the capacity for molecular genetic research in Africa, there are ongoing difficulties (Martin et al. 2022). These include the need for expensive laboratory equipment to process samples which even if donated would be difficult to maintain. The absence of ancillary facilities to utilize these equipment (e.g., stable electricity, technical expertise and reliable internet connection) in many African countries. As research collaborations get stronger between low- and middle-income versus high-income countries, some of these challenges may be overcome, however, this is likely to take several years (Martin et al. 2022).

## Twin Research in Nigeria at Present

A complementary effort would be to look within Africa and utilize existing locally available resources to further promote genetic research. Africa as a continent has the highest rate of twinning in the world (17/1000 births) (Monden et al. 2021) especially along a belt running south-eastwards from Guinea in West Africa to Tanzania and the Comoros in Eastern Africa (Smits and Monden 2011). Much higher rates (up to 46.5/1000 births) have been reported in south-western Nigeria (Akinseye et al. 2019) putting this region among those with the highest rates in the world. However, these estimates may be biased by poor record-keeping (Smits and Monden 2011).

At the present time, there are only two twin registries in Africa – one each in Guinea (longitudinal) and Nigeria (cross-sectional) (Bjerregaard-Andersen et al. 2013; Hur et al. 2013). The Nigerian Twin and Siblings Registry (NTSR) was initiated in 2010 and initially comprised 1538 adolescents (twin individuals and siblings) recruited between 2010 and 2012 (Hur et al. 2013). Between 2013 and 2014, a further 3658 twin adolescents and siblings were recruited (Hur et al. 2019). Of the two African registries, the NTSR has been the more prolific (Hur et al. 2019), producing several papers on neurocognitive and behavioral phenotypes among Nigerian adolescents. However, to date, only one has focused on a mental health phenotype – internalising problems (Oginni & Hur, 2022). The cross-sectional nature of the NTSR also means changes during earlier development cannot be assessed but the success of the NTSR can be taken as a proof of concept i.e., that twin studies are viable in Nigeria.

## The Future of Mental Health Twin Research in Nigeria

An important next step would, therefore, involve making twin studies in Nigeria more efficient. In its most basic form, the classical twin design estimates genetic and (shared and individual-specific) environmental influences on individual differences by comparing similarities in monozygotic and dizygotic twin pairs raised together. This design has been explained in detail by previous authors (Boomsma et al. 2002; Rijsdijk and Sham 2002; Willoughby et al. 2023). The utility of the twin design in Nigeria can be increased by recruiting younger twins and following them up over time to investigate changes in genetic and environmental influences on mental health during development (e.g., Rimfeld et al. 2019). Other relatives such as the twins’ parents and later on, their children, can also be included to extend this method to investigate genetic and environmental influences on parent-child similarities (Keller et al. 2010; McAdams et al. 2018). Considering the ongoing developments in molecular genetics in the continent (Martin et al. 2022), it would hopefully soon become possible to incorporate genomic data to test causal hypotheses (Minică et al. 2018; Oginni et al., 2023a, 2022; Singh et al., 2023) about the etiology of mental health problems in an African country.

Specifically, potential benefits of twin studies to mental health research and clinical practice in Nigeria can be conceptualized at the three levels of care – prevention, treatment and rehabilitation (Ademiluyi and Aluko-Arowolo 1990). With respect to prevention, gene-environment interaction models (Purcell 2002) can be used to identify modifiable environmental influences which increase genetic influences on psychopathology and can be targeted for preventive interventions (e.g., Oginni & Hur, 2022). Causal twin models such as the Direction of Causation model (Heath et al. 1993) or the Mendelian Randomization-Direction of Causation model which incorporates genomic information (Minică et al. 2018; Oginni et al. 2023b) can be used to identify causal links between environmental risk and psychopathology outcomes. The mechanisms of these causal links (i.e., mediators or moderators) can also be investigated to identify specific targets of preventive/therapeutic interventions (Oginni et al., 2023a). Such causal models can also inform rehabilitation by demonstrating causal links between psychopathology and adverse psychosocial outcomes, as well as the mechanisms of these. From a developmental perspective, longitudinal twin designs are useful for investigating how genetic/environmental/causal influences on psychopathology change during critical developmental periods like puberty (Nivard et al. 2015). Such critical points could indicate periods of increased vulnerability to risk exposures or sensitivity to protective influences which can, in turn, inform the timing of proposed intervention. A less recognized potential of twin analyses is the extension of findings from randomized controlled trials (Sumathipala et al., 2017) which are uncommon among children and adolescents especially in low- and middle-income countries (Klasen and Crombag 2013). Randomising identical twins to different intervention groups during a controlled trial (e.g., using a cotwin-control design) can control for genetic confounding and increase statistical power compared to using singletons (Sumathipala et al. 2018). The twin design can also be used to control for pre-existing genetic and environmental influences while testing whether therapeutic effects of interventions reflect novel genetic and/or environmental influences e.g., a within-individual control design using a Cholesky decomposition (Haworth et al. 2016). Finally, twin studies can be used to examine the mechanisms of comorbidities. In high-income countries, such analyses have helped inform the categorization of psychiatric disorders e.g., the specification of the Obsessive Compulsive and Related Disorders cluster in the ICD-11 (e.g., Monzani et al. 2014; Stein et al. 2016). In Nigeria, such analyses can be applied to identify comorbid psychopathologies that may benefit from common interventions (i.e., common genetic and environmental influences), or mechanisms of comorbidities between physical childhood conditions and psychopathology risk. While these findings can be beneficial for reducing the large mental health gap in Nigeria and other low- and middle-income countries; they may also highlight novel mechanisms that can be useful for global mental health research and clinical practice.

We note that the twin design is not without its limitations. These include the violation of underlying assumptions such as twin pairs being treated equally similarly irrespective of their zygosity, random mate selection, and independent genetic and environmental influences (Rijsdijk and Sham 2002). However, extensions of the classical twin design are able to adjust for any violations of these assumptions and yield less biased estimates of genetic and environmental influences (e.g., Boomsma et al. 2010). Thus, initiating a longitudinal and well-designed twin study in Nigeria along with increasingly available molecular genetic technology will position Nigeria to take advantage of the emerging statistical expertise to innovatively answer complex questions about the etiology of mental illnesses in a low- and middle-income country.

Having set out some of the opportunities for twin research in Nigeria, we next discuss potential challenges and proffer solutions to overcoming them.

### Challenges to Twin Research in Nigeria

#### Recruitment

Twins can be identified from school and hospital records, and media adverts (newspaper, radio or television; Odintsova et al. 2018). Potential difficulties include the use of paper for storing information which can easily be misplaced. Records in many low- and middle-income countries are also not routinely updated, hence, contact details of households with twins may be outdated and participants unreachable. Primary and secondary schools can be an alternative strategy for reaching older twins and this would require planning in advance and collaboration with the relevant government ministries. While primary school education is free (in public schools) and compulsory, attendance is not enforced (Abdullahi and Abdullah 2014) and enrolment can be unpredictable; which can make it difficult to prioritize schools where twins may be identified (Hur et al. 2017). Children from higher socioeconomic classes may also be more likely to attend school which may make school-based samples unrepresentative. With respect to adverts, newspapers may not be very useful considering the low rates of reading newspapers (e.g., Apuke and Omar 2020) and relatively low literacy rates (60% in 2018 and 51% in 2008; Nielsen 2021). There are up to 117 radio and 103 television broadcasting corporations in Nigeria (Babawale 2018; Hendrickson 2022). This means that a working knowledge of the local consumer pattern is needed in the desired regions of research.

### Data Collection

Actual data collection can be via face-to-face interviews, telephone calls, virtual meetings or surveys or questionnaires, disseminated via the post. The latter three approaches can reduce the need for physical travel but they may not facilitate the establishment of rapport and this can be potentially worsened by the high prevalence of phone and online scams (Jack and Ene 2016). Postage in Nigeria is further unreliable with delivery facing varying degrees of delay and few Nigerians registering for postage delivery. Face-to-face interactions may be better for establishing rapport, gaining trust and verifying the identities of the research participants to increase the validity of the data. However, these will often require travel and time off work which may be difficult for those who are not self-employed or who have low incomes.

### Analyses

One potential limitation to twin research in Nigeria and many other low- and middle-income countries is the low level of technical expertise in twin data analyses. As an example, cross-sectional twin data on ≈ 1600 Nigerian twin pairs has been available for collaborations since 2012 (Hur et al. 2019). However, it was only in 2022 that a Nigerian initiated a collaboration using the dataset (Oginni & Hur, 2022) having developed expertise in the method.

## Dissemination and Translation of Research Findings

Health research findings in Nigeria tend to be disseminated mostly within academia e.g., scientific conferences and peer-reviewed journal publications. Specific to mental health research, it is possible that this reflects the largely supernatural explanation for mental illness in the general public especially among those with lower levels of education (Adebowale and Ogunlesi 1999; Adewuya and Makanjuola 2008). However, the low level of public discourse on mental health may also reflect a lack of political will which manifests as underfinancing of healthcare services and research generally (Abubakar et al. 2022). This apparent apathy may reflect competing health, economic and social needs; inadequate planning and corruption (Abubakar et al. 2022). Although, with the proliferation of relevant non-governmental (civil society) organizations in the mental health space, this may be improving.

### General/Systemic Challenges

Caution should be exercized in including very young twins (e.g., infants) in a longitudinal study. This is based on the relatively high under-5 mortality rate in Nigeria − 132/1000 births which ranges between 62/1000 births in the South-West zone to 187/1000 in the North-West zone (National Population Commision 2018). However, mortality is typically highest within the first year of life (National Population Commision 2018). Internal migration between different regions of the country may also increase the risk for attrition in longitudinal studies. Political and economic instability (Baah et al. 2023), and intermittent inter-ethnic and religious conflicts (Hassan 2023; Salawu 2010) may increase insecurity which may not facilitate research. Local activities such as traditional market days (i.e., days when traders from surrounding areas converge on a single market at weekly or fortnightly intervals to sell their wares; Daniel and Ezema 2018) may be attractive to community members including traders who will then not be available to participate in research at such times.

### Potential Solutions for Challenges to Twin Research in Nigeria

#### Recruitment

This can be facilitated by simultaneously combining multiple strategies including hospital records, schools and adverts. However, the use of adverts should be preceded by preliminary investigation of the local patterns of radio and television viewership so that relevant media outlets are targeted. Television and/or radio talk shows about the importance and utility of twin research can also be carried out in the weeks leading up to the study to increase public interest and participation. Considering the relatively high prevalence of twins and the high traditional status accorded twins in south-western Nigeria (Leroy et al. 2002), households with twins are often well-known within communities and to other households with twins. Thus, snowballing will be a useful adjunctive recruitment strategy, especially in south-western Nigeria. Participant details should also be verified and updated at each contact to include contacts of extended family members who are less likely to move away from the study region (e.g., grandparents) to increase retention. Alternative means of contact can also be obtained such as social media handles especially among younger twins (e.g., adolescents).

Another recruitment strategy that has shown some promise is Patient Participant Involvement and Engagement (PPIE) to inform the design and conduct of twin research which we have done preliminarily. First, we identified and collected the phone numbers of 897 mothers of twins from birth and vaccination records in 5 hospital units in three towns in Osun state, Southwestern Nigeria (Ile-Ife, Ilesa and Osogbo; ethical approval was obtained from the Ethics and Research Committee, Obafemi Awolowo University Teaching Hospitals Complex, Ile-Ife, Nigeria). From contacting 256 of these mothers, we established a response rate of 31.6% (n = 81; some phone numbers were no longer active, a few were no longer resident in the region and a minority did not wish to participate). Of those available, we identified three mothers from Ile-Ife and Ilesa (rural/semi-urban settings) who were willing to serve as public contributors (i.e., members of the target population who will help shape the design and implementation of research through their lived experiences through PPIE activities; Health and Care Research Wales 2022). We summarise their perspectives on the different aspects of carrying out a twin-family in the community and factors that will promote participation in Table 1. The public contributors (and traditional birth attendants - community-based midwives) indicated snowball sampling was a viable recruitment strategy as they (and other mothers of twins) knew other mothers in the community. Notably, the public contributors emphasized the use of radio rather than television for advertisement and dissemination as more households used the former. Similarly, none of the public contributors recommended the use of social media which may reflect their sociodemographic profile; however, this should be further investigated. Hospitals were recommended as the site of data collection and dissemination activities to facilitate credibility of the researchers. The public contributors also gave suggestions for compensation and other incentives for research participation. Although, we did not assess their willingness to participate in the interpretation of research findings, we believe their participation in this and other research activities (including questionnaire translation and dissemination of results) can promote a sense of research ownership, and interest in using the research findings. PPIE activities are innovative in Nigeria and, specific to mental health, can additionally help in reducing mental health stigma in the community.

**Table 1:** Summary of the Challenges and their Solutions for Twin Research in Nigeria and Feedback from Public Contributors in Osun state, Nigeria

	Challenges	Solutions/opportunities	Patient Participant Involvement/Engagement
Recruitment	• Hospital records not routinely updated. • Suboptimal/unpredictable/biased school enrolment and attendance. • Low rates of reading newspapers. • Multiple television and radio stations.	• Multiple recruitment approaches can be combined. • High rates of twinning and celebrated status of twins can facilitate snowballing. • Preliminary assessment of television and radio consumption patterns before placing adverts. • Community involvement and engagement.	• Mothers of twins (Public contributors) and community-based traditional birth attendants* knew other mothers of twins; and were willing to help invite mothers of twins. Hence snowball sampling is a viable recruitment strategy. • Public contributors advised using radio adverts and doing this in advance of the actual study to increase credibility.
Data collection	• High rates of internet/phone-mediated fraud. • Poor postage services. • Direct and indirect costs of travel.	• Face-to-face interactions may facilitate trust. • Linkage with established health institutions can facilitate trust for non-face-to-face interactions. • Monetary compensation for travel.	• Public contributors recommended collecting data at government hospitals to facilitate credibility rather than home visits as they be unavailable due to work or other social/religious commitments. • Public contributors recommended cash compensation for time and transport costs (especially the latter which had recently increased); and tangible items which can be useful for the children e.g., water-bottles for school. • Public contributors expressed more comfort with providing saliva compared to blood samples. • Incorporating physical health checks into study can serve as a further incentive for participation.
Analyses	• Low technical skill.	• Involvement in collaborative twin research activities. • Investigate phenotypes of interest to local researchers. • Participation in training workshops. • Facilitate conference attendance.	
Dissemination	• Mental health stigma. • Non-scientific model for explaining mental illness in the general public.	• Dissemination and pre-engagement publicity can help reduce mental health stigma. • Can be facilitated by working with existing structures e.g., non-governmental organizations, government parastatals.	• Public contributors were willing to attend dissemination meetings in the hospital. • Public contributors also recommended dissemination via radio programs
General/systemic	• Political/economic instability. • Insecurity. • Local activities which may threaten research participation.	• Careful timing of research activities to avoid periods of unrest. • Targeting research to calmer and more secure regions with less internal migration.	

*Midwives based in the community and often affiliated to religious organizations who supervise deliveries in the community

### Data Collection

As with recruitment, multiple strategies can also be combined. For example, where phone numbers have been obtained from schools or hospital records, households can be contacted by phone, eligibility ascertained, and face-to-face appointments set up in research offices within hospitals and universities. We recommend known institutions as study sites to increase trust in the authenticity of the study among potential participants. Compensating participants adequately for their time and travel costs would also encourage participation. However, considering the high levels of poverty in Nigeria (Baah et al. 2023), a balance needs to be drawn between sufficient compensation for transport and lost income (especially if parents will be involved; otherwise they would not participate) and inducement for fraudulent participation. One potential solution is to verify the eligibility status of potential participants using identification documents such as birth certificates and photographs of twins. Alliances can also be established with trusted members of the community who can help verify the eligibility of the participants. To minimize the need for participants’ travel, alternative arrangements can be made such as collecting the data within the community e.g., at marketplaces. However, eligibility will need to be scrutinized as earlier described.

### Analyses

Avenues to increase the capacity of Nigerian researchers in all aspects of twin research should be maximized. These can include maximising opportunities for research collaborations at all stages of twin research, facilitating training in twin research methodology (as is being done with GINGER for genomic research), subsidising costs of attending international twin research courses (as was advertised for the 2023 International Statistical Genetics Workshop). However, courses for researchers from low- and middle-income settings in Nigeria may need to be initially pitched at introductory levels. Conferences can also serve to expose Nigerian (and other low- and middle-income) researchers to the applications of twin research. Collaborations can also be facilitated by investigating phenotypes that are useful to Nigerian researchers. For example, mental health phenotypes would have more practical relevance to clinicians who carry out most of the mental health research in Nigeria compared to relatively abstract phenotypes like height (Silventoinen et al. 2003). This may reflect a more utilitarian (problem-based) rather than curiosity-driven approach to research in low- and middle- versus high-income countries respectively (Jaffe et al. 2020). However, this difference in priorities may be overcome by balancing the interests of collaborating parties.

### Dissemination

This should be targeted at both the lay public including community leaders, parents of twins and twins themselves, and specific stakeholders such as officials of the ministries of health and education for implementation. As before the study commencement, radio and/or television talk shows can be used to disseminate research findings with question-and-answer sessions. These can be complemented by using other internet-based social media as may be relevant. However, more targeted dissemination meetings can be held to involve the relevant ministries with the results and actionable outcomes communicated as simply as possible. Other activities used in established twin cohorts such as engagement with social media and competitions for twins can also be useful especially for longitudinal twin studies. Together, these activities can potentially increase public discourse of mental health and reduce associated stigma. In both the shorter and longer term, researchers can work with non-governmental organizations and thought leaders such as religious leaders to improve the dissemination of knowledge from genetically-informed mental health research. This approach has helped with the response to the HIV/AIDS epidemic whereby researchers and physicians, non-governmental (including religious) organizations partnered with the health ministries to provide research-based public education and interventions alongside regular surveys to monitor the progress of the HIV epidemic alongside ongoing interventions (Adeyi et al. 2018). Though targets still have yet to be met, significant progress is being made.

### General Considerations

Longitudinal twin research can, for now, focus on older twins (e.g., two years and above) in regions with relatively better childhood survival (National Population Commision 2018) to minimize attrition due to infant mortality. Regions with more stable populations can be targeted for longer term research e.g., the south-west versus the south-east (Tacoli and Mabala 2010). Large scale twin researchers in Nigeria will need to keep abreast of the socio-political climate, avoiding periods that may be associated with turmoil e.g., national or local elections. The research environment will also need to be carefully considered and may involve initially focusing on more politically stable and peaceful regions with relatively higher twinning rates like south-western Nigeria. Public engagement activities can help identify periods when participation is likely to be low and such periods used for alternative research activities.

## Conclusion

Considering the high rates of twinning in Nigeria, twin research can serve as a readily available and relatively affordable approach to domesticating genetic research in Nigeria. We have highlighted possible challenges and suggested means of overcoming these as well as existing opportunities. We stress that twin research is not an alternative to other population-based statistical genetic studies. Rather, these methods can be combined complementarily and innovatively, both now and in the future, to further increase the global understanding of the complex etiological mechanisms of mental health problems.
